# Paraneoplastic Arthritis Mimicking Late-Onset Rheumatoid Arthritis in an Older Smoker: A Diagnostic Challenge

**DOI:** 10.7759/cureus.103360

**Published:** 2026-02-10

**Authors:** Narayana Swamy, Saraswathi Saiprasad, David Garate, Kristi Kway

**Affiliations:** 1 Rheumatology, Baylor Scott &amp; White All Saints Medical Center - Fort Worth, Fort Worth, USA; 2 Endocrinology, Diabetes, and Metabolism, Baylor Scott &amp; White All Saints Medical Center - Fort Worth, Fort Worth, USA; 3 Dermatology, University of Texas Medical Branch at Galveston, Galveston, USA; 4 Internal Medicine, Baylor Scott &amp; White All Saints Medical Center - Fort Worth, Fort Worth, USA; 5 Internal Medicine, Texas Christian University (TCU) School of Medicine, Fort Worth, USA

**Keywords:** chronic inflammatory polyarthritis, interstitial lung disease, lung cancer, paraneoplastic arthritis, rheumatoid arthritis mimic, rheumatoid factor, smoking, usual interstitial pneumonia

## Abstract

Paraneoplastic rheumatic syndromes may present with inflammatory arthritis that closely mimics rheumatoid arthritis (RA), leading to diagnostic delay. We describe an older male with progressive inflammatory polyarthritis and a low-titer positive rheumatoid factor (RF), initially diagnosed with late-onset seropositive RA. Although his arthritis improved with glucocorticoids and hydroxychloroquine, persistent respiratory symptoms prompted further evaluation. High-resolution computed tomography (HRCT) of the chest revealed progressive fibrotic interstitial lung disease (ILD) with an indeterminate usual interstitial pneumonia (UIP) pattern and multiple pulmonary nodules suspicious for malignancy. Subsequent biopsy confirmed synchronous non-small cell lung carcinoma. The rheumatologic diagnosis was revised to paraneoplastic arthritis mimicking RA. This case highlights the importance of maintaining a high index of suspicion for occult malignancy in older patients presenting with new-onset inflammatory arthritis, particularly in smokers with pulmonary symptoms.

## Introduction

Paraneoplastic syndromes are systemic manifestations of malignancy that occur independently of direct tumor invasion or metastasis. Lung cancer is among the malignancies most commonly associated with paraneoplastic phenomena, including rheumatologic manifestations such as inflammatory arthritis [1,2]. Paraneoplastic arthritis, also termed carcinomatous polyarthritis, is a rare but important entity that may precede the diagnosis of malignancy and frequently resembles rheumatoid arthritis (RA) in its clinical presentation [3]. Paraneoplastic arthritis often differs from classical RA by its abrupt onset, relative lack of erosive joint damage, poor or transient response to disease-modifying antirheumatic therapy, and frequent association with systemic features such as weight loss or constitutional symptoms. 

Late-onset rheumatoid arthritis (LORA), typically defined as RA with onset after age 60, presents unique diagnostic challenges. In older adults, inflammatory arthritis may reflect alternative etiologies, including infection, crystal disease, or malignancy. While rheumatoid factor (RF) positivity may support a diagnosis of RA, RF lacks specificity and may be present in chronic inflammatory states, infections, and malignancy [4]. Recognizing these mimics is essential to avoid diagnostic anchoring and delays in identifying underlying cancer.

## Case presentation

A Hispanic man in his 70s with a long-standing history of chronic cigarette smoking presented for rheumatologic evaluation with a three-month history of progressively worsening joint pain. He reported pain and stiffness involving multiple peripheral joints, including the bilateral metacarpophalangeal (MCP) and proximal interphalangeal (PIP) joints, wrists, shoulders, and knees. Pain severity was rated 6/10 and was associated with early-morning stiffness lasting more than 60 minutes, and symptoms were exacerbated by prolonged inactivity and exposure to cold air. He denied fever, rash, dysuria, or a personal history of psoriasis. Prior treatment with nonsteroidal anti-inflammatory drugs provided only mild relief.

Review of systems was notable for a chronic dry cough. He denied chest pain, constitutional symptoms, mucocutaneous lesions, neurologic complaints, gastrointestinal symptoms, or genitourinary symptoms.

On physical examination, the patient appeared well nourished and in no acute distress. Pulmonary examination demonstrated normal respiratory effort, symmetric aeration, and clear breath sounds bilaterally, with no wheezes or rales. Cardiovascular examination revealed a regular rate and rhythm with normal heart sounds. No peripheral edema or skin rashes were present.

Musculoskeletal examination demonstrated symmetric swelling and tenderness of multiple metacarpophalangeal joints and proximal interphalangeal joints bilaterally. The wrists and elbows exhibited a full range of motion without swelling or tenderness. The shoulders demonstrated a mildly restricted active range of motion bilaterally. Hips had a full range of motion. The knees were cool to the touch, without effusion, and had full flexion. Neurologic examination showed the patient to be alert and oriented, with normal speech and intact memory.

Initial laboratory evaluation revealed elevated inflammatory markers and a low-titer positive rheumatoid factor of 21 IU/mL (reference range <14 IU/mL) and a negative anti-cyclic citrullinated peptide (anti-CCP) antibody level of <0.5 U/mL (reference range <3.0 U/mL). Hand radiographs obtained approximately one month before presentation demonstrated generalized joint space narrowing involving the MCP and interphalangeal joints. Laboratory studies obtained at that time and during the initial evaluation are summarized in Table 1.

**Table 1:** Inflammatory and Serologic Laboratory Results Abbreviations: Anti-CCP, anti–cyclic citrullinated peptide antibody; ESR, erythrocyte sedimentation rate; CRP, C-reactive protein; H, high.

Test	Reference Range	2 Months Prior to Initial Evaluation	At Initial Rheumatology Evaluation
Rheumatoid Factor (IU/mL)	<14	16 (H)	21 (H)
Anti-CCP IgG (U/mL)	<3.0	Not obtained	<0.5
ESR (mm/hr)	0–20	78 (H)	34 (H)
CRP (mg/dL)	<0.5	2.5 (H)	3.2 (H)

Based on the patient’s age, symmetric inflammatory arthritis, serologic findings, and radiographic changes, late-onset seropositive rheumatoid arthritis was considered the leading diagnosis. Additional diagnostic considerations included chronic crystal arthropathies, such as chronic polyarticular gout and calcium pyrophosphate deposition disease.

Given the presence of chronic cough in the context of inflammatory arthritis and an extensive smoking history, pulmonary pathology was included in the differential diagnosis. While chronic obstructive pulmonary disease was considered, interstitial lung disease was also contemplated. Oral prednisone 15 mg daily was initiated.

At a two-week follow-up visit, the patient reported marked improvement in joint pain and morning stiffness. Hydroxychloroquine 200 mg twice daily was initiated as disease-modifying therapy, and prednisone was gradually tapered. His inflammatory arthritis remained well controlled over the ensuing weeks.

**Figure 1:**
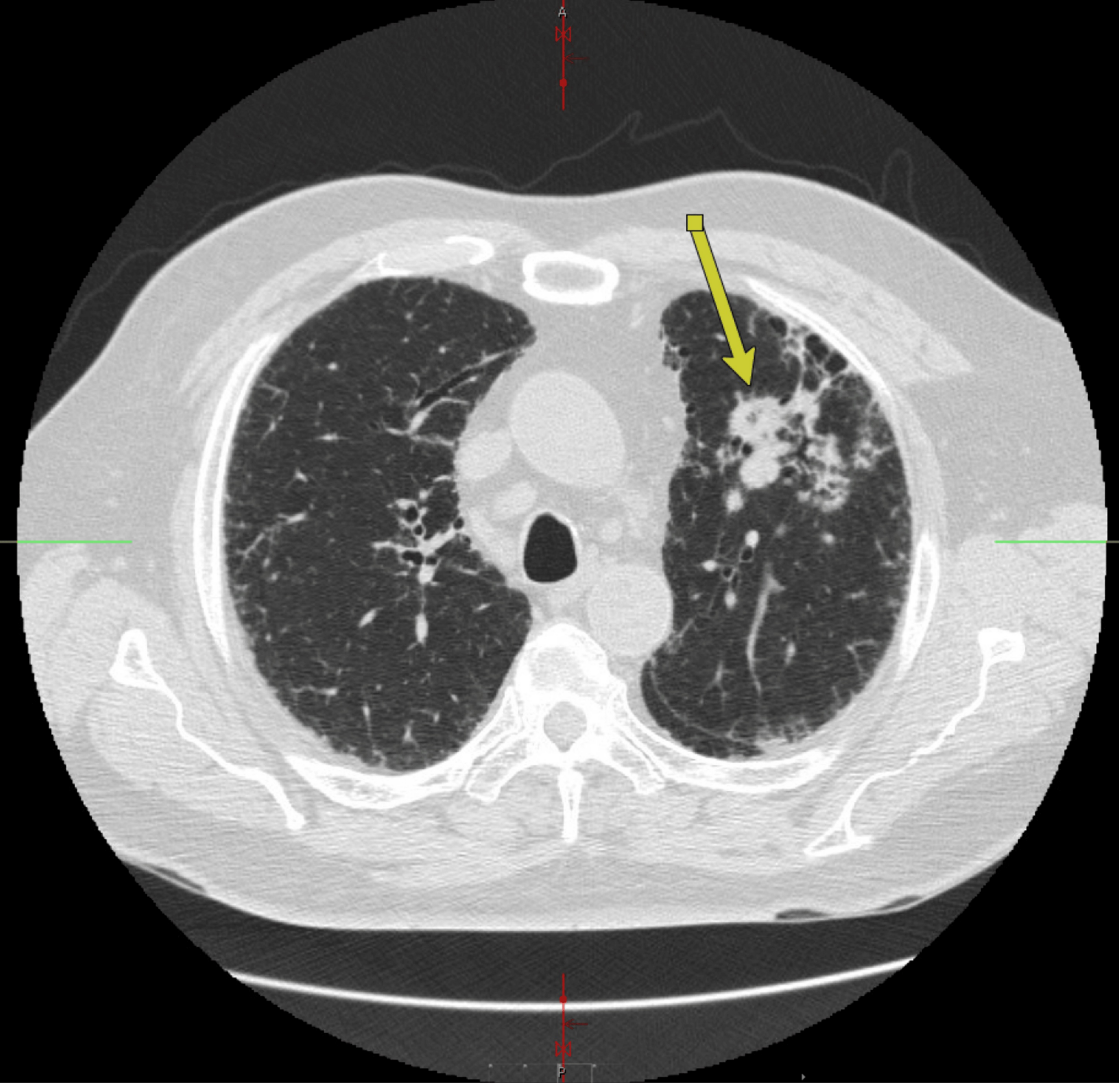
High-resolution CT Chest: conglomerative solid mass-like opacity/clustered nodular opacities in the anterior left upper lobe

**Figure 2:**
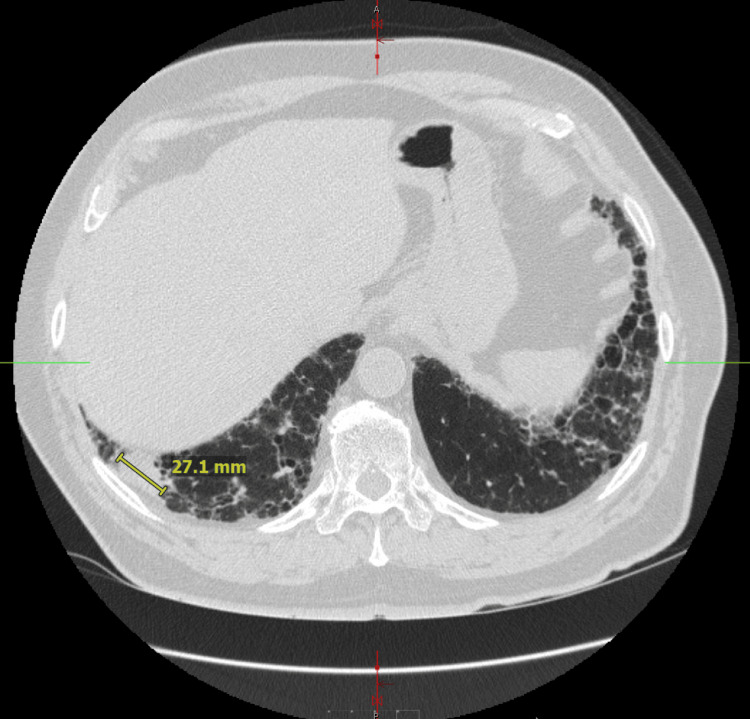
High-resolution CT Chest: 2.7 cm solid nodule in the posterior basal right lower lobe

Despite sustained improvement in musculoskeletal symptoms, the patient continued to experience a persistent cough and progressive dyspnea. A previously recommended high-resolution computed tomography (HRCT) scan of the chest was delayed due to financial constraints and was ultimately obtained approximately four months after initial presentation. HRCT demonstrated progressive fibrotic interstitial lung disease with features suggestive of a usual interstitial pneumonia pattern, along with multiple pulmonary nodules and mass-like lesions. Findings included a conglomerative mass in the anterior left upper lobe (Figure 1) and a 2.7-cm solid nodule in the posterior basal segment of the right lower lobe (Figure 2), raising concern for an underlying malignancy.

The patient was urgently referred to pulmonology. Further evaluation revealed bilateral lung masses with associated mediastinal lymphadenopathy, findings highly suspicious for malignancy given his extensive smoking history. Bronchoscopy with tissue biopsy was initially non-diagnostic, although atypical cells were identified. Infectious etiologies were considered, and QuantiFERON-TB Gold testing returned positive; however, a comprehensive infectious evaluation, including assessment for active tuberculosis and other infections, was negative.

Given persistent concern for malignancy, repeat tissue sampling was pursued. Subsequent biopsy confirmed non-small cell lung carcinoma consistent with adenocarcinoma. The patient was diagnosed with stage IV lung cancer and referred to oncology, where he underwent radiation therapy and initiated palliative chemotherapy.

In light of the malignancy diagnosis, the patient’s inflammatory arthritis, initially classified as late-onset rheumatoid arthritis, was reclassified as paraneoplastic arthritis mimicking rheumatoid arthritis (Table 2).

**Table 2:** Features supporting paraneoplastic arthritis This table summarizes the key clinical, serologic, radiographic, and diagnostic features observed during the patient’s evaluation. “Persistently elevated” refers to abnormal inflammatory marker values documented on multiple measurements over the disease course.

Feature	Findings	Reference range (if applicable)
Age at onset	Late 70s	Not applicable
Rheumatoid factor	Low titer positive (16-21 IU/mL)	<14 IU/mL
Anti-cyclic citrullinated peptide (anti-CCP) IgG	<0.5 U/mL	<3.0 U/mL
Erythrocyte sedimentation rate (ESR)	Persistently elevated (34–78 mm/hr)	0–20 mm/hr
C-reactive protein (CRP)	Persistently elevated (2.5–3.2 mg/dL)	<0.5 mg/dL
Hand radiographs	Joint space narrowing without erosions	Not applicable
Response to corticosteroids	Marked clinical improvement	Not applicable
Pulmonary findings	Bilateral lung masses with mediastinal lymphadenopathy	Not applicable
Final diagnosis	Metastatic lung adenocarcinoma	Not applicable

At one-year follow-up, the patient’s joint symptoms remained well controlled on hydroxychloroquine and very low-dose prednisone, without clinical evidence of active synovitis. He continued a close multidisciplinary follow-up with oncology and pulmonology.

## Discussion

Paraneoplastic arthritis is a rare but clinically important manifestation of malignancy, most associated with lung cancer, and may closely mimic rheumatoid arthritis (RA), leading to diagnostic delay [1-3]. This case illustrates the diagnostic challenges encountered when evaluating new-onset inflammatory arthritis in older adults, particularly in the presence of overlapping serologic and radiographic features. 

Late-onset rheumatoid arthritis (LORA), defined as RA with onset after age 60, often presents diagnostic uncertainty due to comorbid conditions and reduced specificity of serologic markers in this population. Although symmetric polyarthritis and rheumatoid factor (RF) positivity may support an RA diagnosis, RF lacks specificity and may be detected in chronic inflammatory states, infections, pulmonary disease, and malignancy [4]. In this patient, low-titer RF positivity likely contributed to diagnostic anchoring and delayed consideration of alternative etiologies. 

Paraneoplastic arthritis may resemble RA not only clinically but also in its response to therapy. Improvement with glucocorticoids, as observed in this case, does not reliably distinguish RA from malignancy-associated inflammatory arthritis, as tumor-driven cytokine production may be temporarily suppressed by anti-inflammatory treatment [2,3]. Consequently, early symptomatic response should not preclude continued diagnostic vigilance, particularly when systemic features persist. 

The presence of interstitial lung disease (ILD) further complicated the diagnostic evaluation. RA-associated ILD commonly demonstrates a usual interstitial pneumonia (UIP) pattern on high-resolution computed tomography, initially supporting the presumed diagnosis of RA [5,6]. However, the progression of fibrotic lung disease accompanied by multiple pulmonary nodules and mass-like lesions was atypical for uncomplicated RA-associated ILD and prompted further investigation. Ultimately, tissue biopsy confirmed advanced non-small cell lung carcinoma, reframing the inflammatory arthritis as paraneoplastic in origin. 

The patient’s extensive smoking history significantly increased his baseline risk for lung cancer, reinforcing the importance of individualized risk assessment in older adults with inflammatory arthritis [7]. Persistent respiratory symptoms despite improvement in joint manifestations served as a critical clue to warrant multidisciplinary evaluation. Additionally, RF positivity has been described in chronic infections such as tuberculosis, emphasizing the need for cautious interpretation of serologic findings in complex clinical contexts [8]. 

This case underscores the necessity of maintaining a broad differential diagnosis when evaluating inflammatory arthritis in older adults. Persistent systemic symptoms, atypical imaging findings, or discordance between articular and extra-articular disease progression should prompt reassessment for occult malignancy. Early recognition of paraneoplastic rheumatic syndromes enables timely oncologic diagnosis, avoids inappropriate immunosuppression, and facilitates coordinated multidisciplinary care.

## Conclusions

Paraneoplastic arthritis is an important diagnostic mimic of late-onset rheumatoid arthritis in older adults and should be considered even in the presence of low-titer rheumatoid factor positivity. Serologic findings and early symptomatic response to glucocorticoids may be misleading and should not preclude continued evaluation for alternative etiologies, including malignancy. 

Persistent systemic symptoms, progressive pulmonary findings, or atypical imaging abnormalities warrant prompt investigation for occult cancer, particularly in high-risk populations such as chronic smokers. Early recognition of paraneoplastic rheumatic syndromes facilitates timely oncologic diagnosis, prevents inappropriate escalation of immunosuppressive therapy, and enables coordinated multidisciplinary care. Maintaining a high index of suspicion in older patients with new-onset inflammatory arthritis is essential to avoid diagnostic delay and optimize patient outcomes. 
